# CSF3R T618I, SETBP1 G870S, SRSF2 P95H, and ASXL1 Q780* tetramutation co-contribute to myeloblast transformation in a chronic neutrophilic leukemia

**DOI:** 10.1007/s00277-021-04491-2

**Published:** 2021-04-06

**Authors:** Yi Qian, Yan Chen, Xiaoming Li

**Affiliations:** Department of Hematology, Affiliated Hospital of Southwest Medical University, Luzhou, 646000 Sichuan Province China

**Keywords:** Chronic neutrophilic leukemia, CSF3RT618I, AML transformation

## Abstract

Chronic neutrophilic leukemia (CNL) is a rare but serious myeloid malignancy. In a review of reported cases for WHO-defined CNL, CSF3R mutation is found in about 90% cases and confirmed as the molecular basis of CNL. Concurrent mutations are observed in CSF3R-mutated CNL patients, including ASXL1, SETBP1, SRSF2, JAK2, CALR, TET2, NRAS, U2AF1, and CBL. Both ASXL1 and SETBP1 mutations in CNL have been associated with a poor prognosis, whereas, SRSF2 mutation was undetermined. Our patient was a 77-year-old man and had no significant past medical history and symptoms with leukocytosis. Bone marrow (BM) aspirate and biopsy revealed a markedly hypercellular marrow with prominent left-shifted granulopoiesis. Next-generation sequencing (NGS) of DNA from the BM aspirate of a panel of 28 genes, known to be pathogenic in MDS/MPN, detected mutations in CSF3R, SETBP1, and SRSF2, and a diagnosis of CNL was made. The patient did not use a JAK-STAT pathway inhibitor (ruxolitinib) but started on hydroxyurea and alpha-interferon and developed pruritus after 4 months of diagnosis and nasal hemorrhage 1 month later. Then, the patient was diagnosed with CNL with AML transformation and developed intracranial hemorrhage and died. We repeated NGS and found that three additional mutations were detected: ASXL1, PRKDC, MYOM2; variant allele frequency (VAF) of the prior mutations in CSF3R, SETBP1, and SRSF2 increased. The concurrence of CSF3RT618I, ASXL1, SETBP1, and SRSF2 mutation may be a mutationally detrimental combination and contribute to disease progression and AML transformation, as well as the nonspecific treatment of hydroxyurea and alpha-interferon, but the significance and role of PRKDC and MYOM2 mutations were not undetermined.

## Introduction

Chronic neutrophilic leukemia (CNL) is a rare but serious myeloid malignancy. Here, we present a case of CNL and discuss the significance of co-expressed mutations in transformation to acute myeloid leukemia (AML). Our patient was a 77-year-old man and had no significant past medical history and symptoms such as fatigue, weight loss, easy bruising, pruritus, abdominal distention, and night sweats. He was hospitalized with chest pain. There was no splenomegaly and hepatomegaly on physical examination. His complete blood count showed white blood cell (WBC) count 88.90 × 10^9^/L; neutrophil 83.03 × 10^9^/L; red blood cell 3.45 × 10^9^/L; hemoglobin 98 g/L; and platelet 80 × 10^9^/L. Bone marrow (BM) aspirate and biopsy revealed a markedly hypercellular marrow with prominent left-shifted granulopoiesis without dysplasia and blast proliferation. Reticulin fibrosis was not observed. Cytogenetic analysis was normal. Fluorescence in situ hybridization (FISH) results for the JAK2, FGFR1, FIP1L1-PDGFRA, and PDGFRB rearrangement were negative. Polymerase chain reaction results for the BCR-ABL1 fusion, Jak2, MPL, and CALR gene were negative. Next-generation sequencing (NGS) of DNA from the BM aspirate of a panel of 28 genes, known to be pathogenic in MDS/MPN, detected mutations in CSF3R, SETBP1, and SRSF2, and a diagnosis of CNL was made (Tables 1 and 2).

**Table 1:** The mutation outcomes at diagnosis of CNL. Positive mutations

Gene↩	NM	Exon↩	VAF↩
CSF3R↩	NM_000760:c.1853>T(p.T618I)exon 14↩		44.1%↩
SETBPI↩	NM_015559:c.2608G>(p.G870S)exon4↩		47.1%↩
SRSF2↩	NM_003016:c.284C>A(p.95H)exon 1↩		45.8%↩

**Table 2:** The mutation outcomes at diagnosis of CNL. Negative mutations

ASXL1↩	BCOR↩	CALR↩	CBL↩	DNMT3A↩	ETV6↩
JAK3↩	MPL↩	NPM1↩	NRAS↩	PHF6↩	PTPN11↩
RUNX1↩	SF3B1↩	STAG2↩	TET2↩	TP53↩	U2AF1↩
ZRSR2↩	↩	↩	↩	↩	↩

**Fig. 1:**
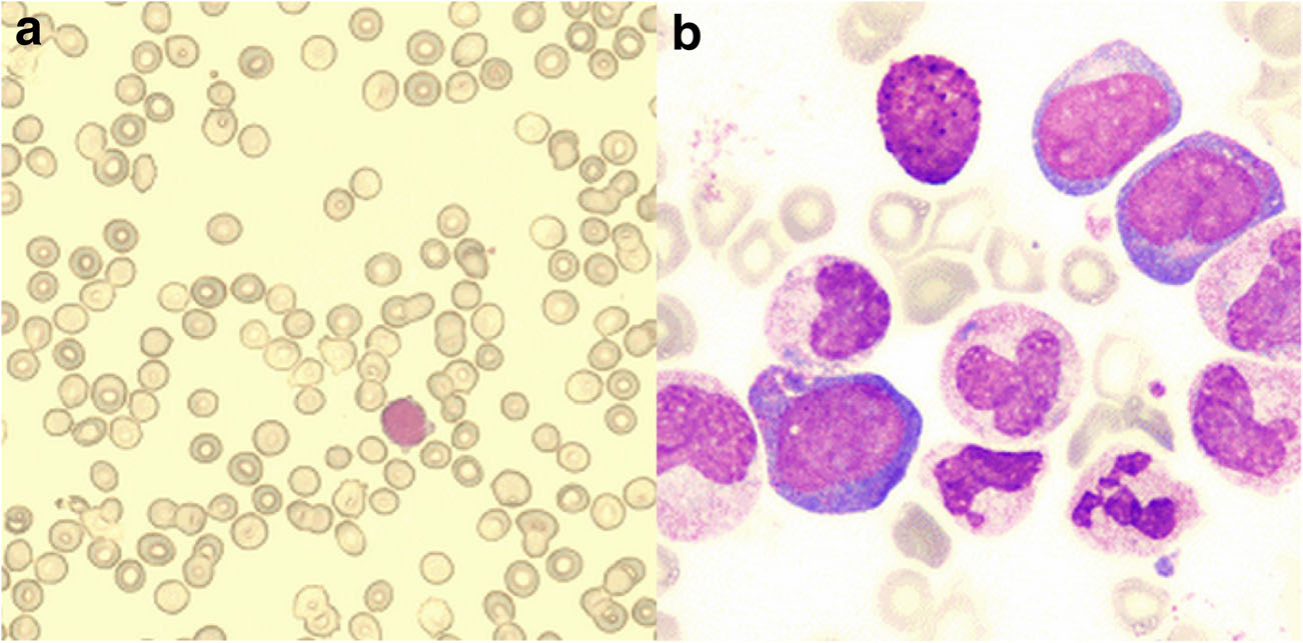
**a** Peripheral blood (Giemsa, ×200). **b** Bone marrow aspirate (Giemsa, ×400)

The patient did not use a JAK-STAT pathway inhibitor (ruxolitinib) for CSF3RT618I mutation but started on hydroxyurea and alpha-interferon to control the myeloproliferation. After 7 days of treatment, he had stabilization of the peripheral blood count, with WBC 7.77 × 10^9^/L, hemoglobin 76 g/L, and platelet 122 × 10^9^/L. But he had no regular follow-up and developed pruritus after 4 months of diagnosis and nasal hemorrhage 1 month later. Serum LDH was 1335.2 U/L. His WBC count increased to 128.52 × 10^9^/L and hemoglobin and platelets dropped to 68 g/dL and 27 × 10^9^/L, respectively. The peripheral blood smear showed 3% myeloblasts (Fig. 1a) and BM aspirate revealed 23.5% myeloblasts and pathological hematopoiesis (Fig. 1b). A diagnosis of CNL with AML transformation was made. We repeated cytogenetic analysis and NGS. Cytogenetic analysis still was normal. FISH results for the BCR-ABL1, ASS1, and +8 were negative. Using the sequencing panel with 96 genes associated with hematopoietic and lymphoid tissue tumors, three additional mutations were detected: ASXL1, PRKDC, MYOM2; variant allele frequency (VAF) of the prior mutations in CSF3R, SETBP1, and SRSF2 increased (Tables 3 and 4). He was admitted for pruritus and nasal hemorrhage, developed intracranial hemorrhage, and expired.

**Table 3:** The mutation outcomes at AML transformation. Positive mutations

Gene↩	NM↩	Exon↩	VAF↩
ASXL1↩	NM_015338:c.2338C>T(p.Q780*)exon12↩		46.1%↩
CSF3R↩	NM_000760:c.1853C>T(p.T618I)exon14↩		49.6%↩
SETBP1↩	NM_015559:c.2608G>A(p.G870S)exon4↩		48.6%↩
SRSF2↩	NM_003016:c.284C>A(p.P95H)exon1↩		49.7%↩
PRKDC↩	NM_006904:c.9921+1G>T exon70↩		4%↩
MYOM2↩	NM_003970:c.3901T>C(p.Y1301H)exon33↩		51%↩

**Table 4:** The mutation outcomes at AML transformation. Negative mutations

ABCB1	ABL1	ANKRD26	APC	ARID1A	ARID1B
ARID2	ATG2B	ATM	ATRX	B2M	BCL10
BCL2	BCL6	BCOR	BCORL1	BIRC3	BLM
BPGM	BRAF	BRCA1	BRCA2	BRIP1	BTG1
BTK	CALR	CARD11	CBL	CBLC	CBLC
CCND1	CCND3	CD28	CD58	CD79A	CD79B
CDKN1A	CDKN2A	CDKN2B	CEBPA	CHD8	CIITA
CREBBP	CRLF2	CSF1R	CTCF	CUX1	CXCR4
DDX41	DIS3	DKC1	DNM2	DNMT3A	EED
EGFR	EGLN1	ELANE	EP300	EPHA7	EPOR
ETV6	EZH2	FAM46C	FAS	FAT1	FBXO11
FBXW7	FLT3	FOXO1	GATA1	GATA2	GFI1
GNA13	GNAI2	GNAS	GNB1	GSKIP	HAXI
HRAS	ID3	IDH1	IDH2	IKZF1	IKZF2
IKZF3	IL7R	IRF4	IRF8	ITPKB	JAK1

In a review of reported cases for WHO-defined CNL, CSF3R mutation is found in about 90% cases [1] and confirmed as the molecular basis of CNL. Concurrent mutations are observed in patients with CSF3R-mutated CNL, including ASXL1, SETBP1, SRSF2, JAK2, CALR, TET2, NRAS, U2AF1, and CBL [2–7]. Elliott et al. found that presence of ASXL1 mutation and thrombocytopenia in CSF3R-mutated CNL were independently predictive of shortened survival on multivariable analysis [2]. Two patients with SETBP1-mutated and ASXL1-unmutated developed AML transformation, whereas two other patients with ASXL1-mutated and SETBP1-unmutated evolved into chronic myelomonocytic leukemia (CMML) in their study. Both ASXL1 mutation and SETBP1 mutation in CNL have been associated with a poor prognosis. SRSF2 mutation has been frequently reported in myelodysplastic syndromes (MDS) and CMML patients which is related to shorter OS and may be considered as an adverse prognostic risk factor in MDS, but not in CMML [8], however, which was undetermined in CNL. In our case, ASXL1 mutation (along with thrombocytopenia) was detected with PRKDC and MYOM2 mutations without the sequencing panel at preliminary diagnosis at low levels, and VAF of CSF3RT618I, SETBP1, and SRSF2 increased at progression to AML transformation. It suggested that concurrence of CSF3RT618I, ASXL1, SETBP1, and SRSF2 mutation may be a mutationally detrimental combination and contribute to disease progression and AML transformation, as well as the nonspecific treatment of hydroxyurea and alpha-interferon, but the significance and role of PRKDC and MYOM2 mutations were not undetermined.

In conclusion, we report here that concurrence of CSF3RT618I, ASXL1, SETBP1, and SRSF2 mutations may co-contribute to AML transformation, but their interactions with PRKDC and MYOM2 mutations in the pathogenesis of CNL as well as their prognostic implications remain to be determined.
